# Comparison of the Interferon-Gamma Release Assay With the Traditional Methods for Detecting *Mycobacterium tuberculosis* Infection in Children

**DOI:** 10.1097/MD.0000000000000087

**Published:** 2014-09-26

**Authors:** Jianwei Zhou, Cui Kong, Yanxi Shi, Zhaocai Zhang, Zhaohong Yuan

**Affiliations:** Clinical Laboratory (JZ, ZZ); Department of Cardiology (CK), Affiliated Hospital of Jining Medical College; Tuberculosis Prevention and Control Institute (YS), Infectious Disease Hospital of Jining City; and Department of Pediatrics (ZY), Affiliated Hospital of Jining Medical College, Jining, Shandong, China.

## Abstract

The purpose of the article is to compare the whole blood interferon-γ release assay (IGRA) with the traditional methods for detecting *Mycobacterium tuberculosis* (MTB) infection in children.

Fifteen childhood patients with tuberculosis and 15 healthy children were recruited. Sputa samples and venous blood were collected, and according to different procedures, IGRA, sputum smear, colloidal gold assay (CGA), fluorescence quantitation polymerase chain reaction (FQ-PCR), and tuberculosis skin test (TST) were, respectively, performed. Thirty healthy children vaccinated with Bacillus Calmette–Guérin (BCG) were also recruited, and the comparative test was carried out between IGRA and TST.

In all of 15 childhood patients with TB, the positive rates were 86.7%, 20.0%, 26.7%, 40%, and 66.7% in IGRA, sputum smear, CGA, FQ-PCR, and TST, respectively. In the children vaccinated with BCG, the positive rate of IGRA was significantly lower than that of TST (6.7% vs 76.7%). From high to low, the specificities of the five methods were sputum smear (100%), IGRA (86.7%), FQ-PCR (86.7%), TST (40%), and CGA (26.7%). Although the specificities of sputum smear and FQ-PCR were more than or equal to that of IGRA, the relative sensitivities limited their applications in populations of children.

IGRA is a sensitive and specific method, and could be taken as a first choice for detecting MTB infection in populations of children.

## INTRODUCTION

Tuberculosis (TB) is a common infectious disease caused by *Mycobacterium tuberculosis* (MTB), which continues to pose a serious threat to human life worldwide. Pediatric TB internationally represents a major public health concern. The World Health Organization reported about 9 million new cases per year, 11% of which occurred in the children under the age of 15. Children contribute to 3% to 6% of the total TB caseload in developed countries, and it increased to more than 25% in developing countries.^1^ Based on the status, the definition of an adequate management of TB in childhood has become one of the main aspects of the global TB control efforts.^2^

The rapid and accurate diagnosis of MTB-infected individual or patient with TB is the focus of TB control,^3^ and the detection of MTB is one of the most important procedures of diagnosis. Traditionally, there are mainly 5 methods for detecting MTB infection in medical laboratories: sputum culture, sputum smear, tuberculin skin test (TST), colloidal gold assay (CGA), and fluorescence quantitation polymerase chain reaction (FQ-PCR). However, because of the characters of long time, low sensitivity, or low specificity, their applications are limited in clinics.^4–6^ Presently, a novel method named interferon-γ release assay (IGRA) has been developed and applied in the diagnosis of MTB infection.^7^ The mechanism of this method is based on the principle that sensitized T cells, which are produced as a result of exposure to MTB antigens, will secrete interferon-γ (IFN-γ) when they are re-exposed to the similar antigens. Accordingly, the patients with TB may be identified by IFN-γ detection in the serum or the mononuclear cells isolated from peripheral blood samples. Based on the mechanisms, there are 2 assays used in clinical laboratories: QuantiFERON-TB Gold In-Tube test (Cellestis Ltd, Australia) and T-SPOT.TB test (Oxford Immunotec, Oxon, UK).^8^

Theoretically, various methods detecting the infection of MTB may produce differences because of different principles or sample types. According to the former methodological studies,^9,10^ FQ-PCR is the assay with the highest sensitivity and specificity among all the methods. Recently, with the development and application of IGRA, the comparative researches of IGRA with other methods emerged. Kim et al^11^ compared IGRA with TST and found that the positive rate of IGRA was 87.8%, while for TST, it was only 48.8%. Obviously, the sensitivity of the former assay was much higher than that of the latter. In the immunosuppressed patient, Stephan et al^12^ also found the same phenomenon. In a specificity study, the total specificity of IGRA was up to 98%, and moreover, there was no notable difference between the specificities of the populations with and without Bacillus Calmette–Guérin (BCG) (96% vs 99%), while in TST, the total specificity was 77% and those for the 2 populations were significantly different (59% vs 97%). This indicated that there was cross-reaction between TST results for those with BCG vaccination, and there may be a certain number of false-positive results with this method.^13^ In summary, the comparison of the methodological studies related to IGRA exhibited 2 aspects of limitations^1^: most studies focused on the comparison of IGRA and TST, and the sensitivity and specificity of the former was both higher than the latter.^11–14^ There was no report about the complete comparison of IGRA with other methods.^2^ The experimental subjects mainly were the adults, and few reports focused on the evaluation of IGRA in populations of children comparing different methods.^15,16^

In this study, sputum smear, CGA, TST, and FQ-PCR were simultaneously used to detect the MTB infection in populations of children. In the meantime, IGRA, a method that was developed in house, was used to detect the concentration of IFN-γ with enzyme-linked immunosorbent assay (ELISA) (Beijing Wantai Biological Pharmacy Enterprise Co, Ltd, Beijing, China) after incubation of the childhood patient blood with the specific antigen. The methodological comparisons of IGRA with other assays were completely carried out.

## MATERIAL AND METHODS

### Subjects

In accordance with the criteria developed by the Tuberculosis Branch of the Chinese Medical Association, Beijing, China, 15 well-diagnosed children with TB (9 boys and 6 girls, aged 2–14 years) were recruited from the Affiliated Hospital of Jining Medical College or Tuberculosis Prevention and Control Institute, Jining, Shandong, China. Besides, 15 and 30 healthy children were also enrolled according to the different experimental needs: the former should be excluded from TB and the latter should have been successfully vaccinated with BCG. No participant was treated with immunosuppressant, and those with human immunodeficiency virus infection and other immunodeficiencies were excluded. Written informed consents were obtained from the guardians of all participants. The study was approved by the Ethics Committee of the Affiliated Hospital of Jining Medical College.

### Methods

According to the routine procedure, sputa samples were collected to make smear for finding MTB with microscope. One milliliter ethylenediaminetetraacetic acid-anticoagulative and 1 mL nonanticoagulation venous blood were collected from each donor, respectively. In accordance with the manufacturer’s instructions, the former was used to detect MTB antibodies by a colloidal gold method kit (MP Biomedicals Asia-Pacific Pte. Ltd, Singapore) and the latter was used to carry out FQ-PCR (DAAN Gene Co, Ltd, Sun Yat-sen University, Guangzhou, China).

TST was performed as follows: the subjects were intradermally injected 0.1 mL purified protein derivative (PPD) of tuberculin (Chengdu Rongsheng Biological Pharmacy Co, Ltd, Chengdu, China), containing 5 units of tuberculin, in the anterior regions of the forearms. The skin response was assessed after 72 hours, and ≥10 mm of firm swelling at the injection site was considered as positive.

Three milliliter heparinized blood was collected from each of the subjects and divided into 3 tubes, containing the TB antigen, the positive and the negative controls, respectively. Then all the tubes were incubated for 22 ± 2 hours at 37°C. Finally, the supernatant was obtained after centrifugation and used for IFN-γ ELISA.

### Statistical Analysis

The data were analyzed using SPSS15.0 software, SPSS Inc, Chicago, IL. The differences between the quantitative data were analyzed with a χ^2^ test. *P* < 0.05 was considered to be significant.

## RESULTS

### Positive Rate of Different Methods

In all of 15 childhood patients with TB, there were 13, 3, 4, 6, and 10 cases that exhibited positive results in IGRA, sputum smear, CGA, FQ-PCR, and TST, respectively. Accordingly, the positive rates were 86.7%, 20.0%, 26.7%, 40%, and 66.7%. The positive rate of IGRA was significantly higher than those of the other 4 methods (all *P* < 0.05; Tables 1 and 2). The positive rate of TST was less than that of IGRA, but higher than those of sputum smear, FQ-PCR, and CGA, and the differences were also statistically significant (*P* < 0.05; Tables 1 and 2).

**TABLE 1:**
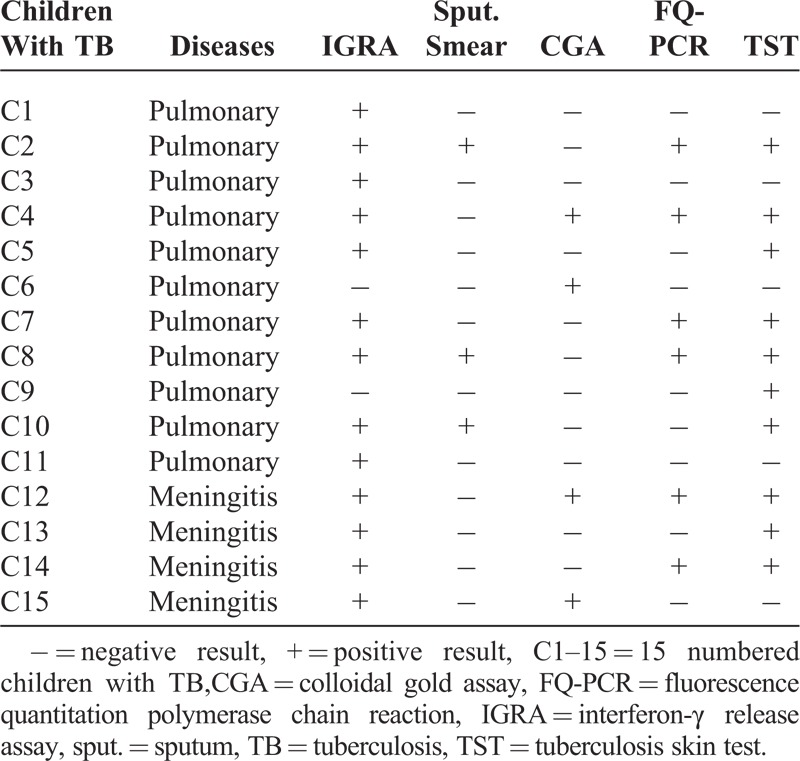
Results of Each Method for Detecting Tuberculosis in Children

**TABLE 2:**
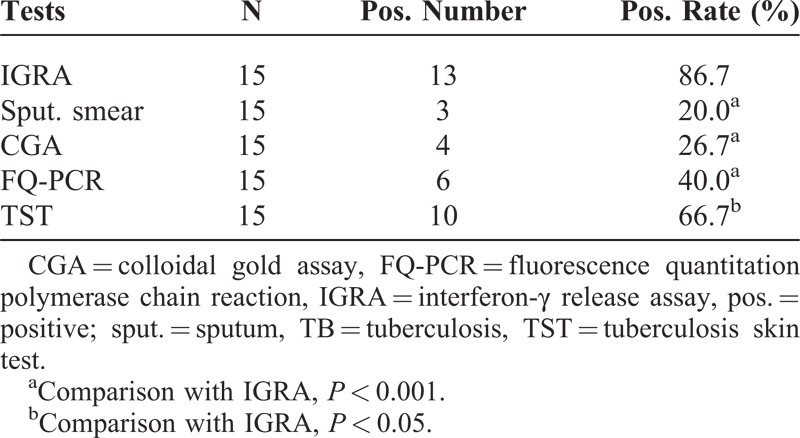
Total Positive Results of Different Methods in the Children With TB

### Effects of BCG Vaccination on IGRA and TST

In order to observe the interference of BCG on IGRA and TST, 30 healthy children successfully vaccinated with BCG were recruited. IGRA and TST were carried out simultaneously. As a result, there were 2 and 23 positive cases in IGRA and TST, respectively; the according ratios were 6.7% and 76.7%, and the difference was significant (*P* < 0.001) (Table 3).

**TABLE 3:**
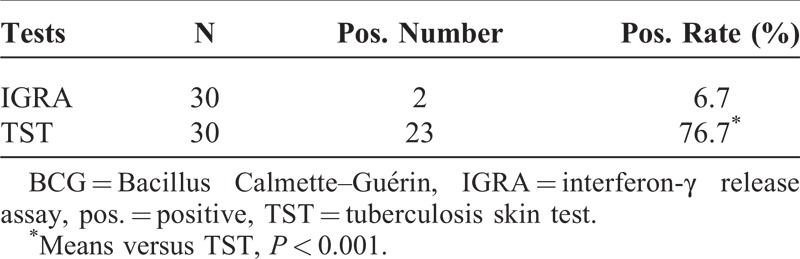
Effects of BCG on IGRA and TST

### Specificity of IGRA and Other Methods

In the 15 healthy children, there were 13, 15, 4, 13, and 6 cases with negative results in IGRA, sputum smear, CGA, FQ-PCR, and TST, respectively. The according negative rates (which were equal to the specificity for each of the methods) were 86.7%, 100%, 26.7%, 86.7%, and 40% (Table 4). There was no difference between the specificities in IGRA, sputum smear, and FQ-PCR (all *P* > 0.05), while the specificity of IGRA was significantly higher than those of CGA and TST, respectively (both *P* < 0.01).

**TABLE 4:**
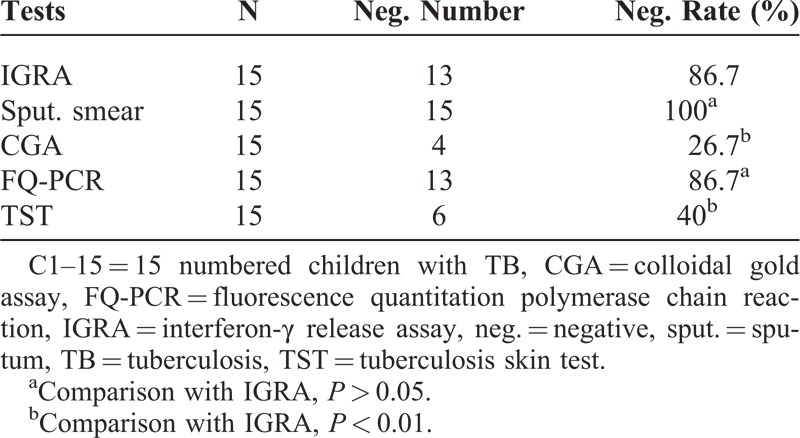
Specificities of the Five Methods

## DISCUSSION

IGRA is a novel method to determine the infection of MTB, and has also been recently accepted as a guideline for diagnosing TB in some countries.^17–19^ In the studies of IGRA application, it is considered as an assay of high sensitivity,^7,8^ and the positive rate is even high to 95% in a report, which was significantly higher than those of the conventional tests, including PPD, MTB antibody, mycobacterial culture, and sputum smear tests.^4^ In this study that included 15 childhood patients with TB, there were 13 cases that exhibited positive results, and the positive result rate was 86.7%, which was lower than the above data. The difference probably is because of the different research subjects, who were not adults but, in this study, were children. In fact, the application of IGRA in pediatric populations is still currently under debate, and the caution was recommended for its usage and interpretation in children.^20^Some authors considered that it should be used as a suboptimal assay in children,^16^ but this opinion has not been approved by others.^21^ This article just focuses on the comparative study of IGRA and other traditional methods.

As shown in Tables 1 and 2, there were 6 positive cases in the sputum smear assay, and the positive rate was 20.0%, which was consistent with the report by Kim et al.^22^ However, the data was significantly lower than that of the IGRA. Given that sputum smear method has a character of low-positive rate with difficulty of collecting specimen and paucibacillary nature of sputum for children patients with TB,^23^ its application is limited in clinical laboratories. Based on these points, IGRA is superior to sputum smear for the detection of MTB infection in childhood populations.

In CGA, there were 4 cases that exhibited positive results, and the positive rate was only 26.7%, which was slightly lower than the report of Liu^24^ and much lower than that of IGRA in this study. The results showed that there may be about 85% of positive cases missed with CGA in populations of children. Therefore, CGA is also more restricted than IGRA in clinics.

FQ-PCR is another usual method for the diagnosis of TB. The samples may be sputum or venous blood. Because the correct sputum is hard to obtain from children, venous blood were collected as the specimen in this study. As shown in the result, the positive rate of the method was 40%, which was alike to the report by Hajiabdolbaghi et al (41.1%).^25^ But comparing with that of IGRA (86.7%), the difference was statistically significant. So based on the sensitivity, FQ-PCR is inferior to IGRA for determining whether the plasma is positive for MTB or not.

According to the current meta-analysis, the sensitivity of TST was about 80% in children with active disease,^15,26^ and it was lower than that in adults^27^; while in this study, in all of the 15 childhood patients with TB, there were 10 positive cases, and the positive rate was only 66.7%, which was significantly lower than that of IGRA. Besides, some reports showed that there was a cross-reaction between TST and BCG vaccination,^28,29^ so the positive relation between the positive results of TST and MTB infection was suspicious. In order to further investigate the effect of BCG vaccination on IGRA and TST, 30 children with successful immunization with BCG were recruited. In the result, the positive rates of IGRA and TST were 6.7% and 76.7%, respectively. This data indicated the strong cross-reaction between TST and BCG, while IGRA exhibited little antigenic cross-reactivity with BCG.^30,31^ So on the aspect of anti-interference of BCG vaccination, IGRA is better than TST for measuring MTB infection.

Additionally, another 15 healthy children were enrolled in this experiment. The IGRA, sputum smear, CGA, FQ-PCR, and TST were simultaneously performed. In the results, the negative rates (which equaled to the specificities of the different methods) were 86.7%, 100%, 26.7%, 86.7%, and 40%, respectively. Obviously, the specificity of IGRA is slightly lower than that of sputum smear and equal to that of FQ-PCR, but higher than those of CGA and TST, respectively. Although the sputum smear and FQ-PCR have relative high specificity, their sensitivities are rather low in children comparing with IGRA. Therefore, based on the data, it can be said that the IGRA is a very specific marker for the diagnosis of childhood patients with TB. However, in several researchers’ opinions, as a predictive marker for progression, the use of IGRA was especially risky in children aged <5 years.^32^However, such a phenomenon is not found in this study; in near future, larger populations will be considered for the further deep investigation.

## CONCLUSIONS

Compared with sputum smear, CGA, FQ-PCR, and TST, IGRA is a method with high sensitivity and specificity. Therefore, within the sample studied herein, IGRA can be taken as a first choice for the detection of MTB infection in populations of children.

## ACKNOWLEDGMENT

The authors thank all the children and their parents who took part in or supported the study.
